# Why we fail: mechanisms and co-factors of unsuccessful thrombectomy in acute ischemic stroke

**DOI:** 10.1007/s10072-020-04244-5

**Published:** 2020-01-23

**Authors:** Dominik M. Heider, Andreas Simgen, Gudrun Wagenpfeil, Philipp Dietrich, Umut Yilmaz, Ruben Mühl-Benninghaus, Safwan Roumia, Klaus Faßbender, Wolfgang Reith, Michael Kettner

**Affiliations:** 1 Department of Diagnostic and Interventional Neuroradiology, Saarland University Hospital, 66421 Homburg/Saar, Germany; 2 Institute of Medical Biometry, Epidemiology and Medical Informatics, Saarland University, Medical Faculty, Homburg/Saar, 66421 Germany; 3 Department of Neurology, Saarland University Hospital, 66421 Homburg/Saar, Germany

**Keywords:** Stent retriever failure, Unsuccessful thrombectomy, Stroke, Intraarterial treatment, Interventional thrombolysis

## Abstract

**Purpose:**

Mechanical thrombectomy (MT) is an effective treatment for patients suffering from acute ischemic stroke. However, recanalization fails in about 16.5% of interventions. We report our experience with unsuccessful MT and analyze technical reasons plus patient-related parameters for failure.

**Methods:**

Five hundred ninety-six patients with acute ischemic stroke in the anterior circulation and intention to perform MT with an aspiration catheter and/or stent retriever were analyzed. Failure was defined as 0, 1, or 2a on the mTICI scale. Patients with failing MT were analyzed for interventional progress and compared to patients with successful intervention, whereby parameters included demographics, medical history, stroke presentation, and treatment.

**Results:**

One hundred of the 596 (16.8%) interventions failed. In 20 cases, thrombus could not be accessed or passed with the device. Peripheral arterial occlusive disease is common in those patients. In 80 patients, true stent retriever failure occurred. In this group, coagulation disorders are associated with poor results, whereas atrial fibrillation is associated with success.

The administration of intravenous thrombolysis and intake of nitric oxide donors are associated with recanalization success. Intervention duration was significantly longer in the failing group.

**Conclusion:**

In 20% of failing MT, thrombus cannot be reached/passed. Direct carotid puncture or surgical arterial access could be considered in these cases.

In 80% of failing interventions, thrombus can be passed with the device, but the occluded vessel cannot be recanalized. Rescue techniques can be an option. Development of new devices and techniques is necessary to improve recanalization rates. Assessment of pre-existing illness could sensitize for occurring complications.

## Introduction

### Background

Mechanical thrombectomy (MT) is a therapeutic option for patients with acute ischemic stroke; it became more important in recent years due to several randomized controlled trials proving better therapeutic outcome compared to intravenous tissue-type plasminogen activator for intravenous thrombolysis (IVT) alone [1–5].

Literature reports success rates of about 83%, whereas 17% of the interventions are not successful [6]. We report our experience with unsuccessful endovascular treatment and analyze technical reasons for failure as well as patient-related parameters, especially medical history.

In our retrospective single-center study, we investigated all treatment protocols of failing interventions and screened those patients for associated pre-existing illness and medication. There are many possible reasons for failure, from unsuccessful vascular access to distal embolization, which are described in recent literature [7, 8].

Elevation of recanalization rates is an important step for improved stroke therapy and better patient outcome [9, 10].

## Material and methods

### Methods and study design

In a retrospective single-center study, 596 patients with acute ischemic stroke of the anterior circulation (internal carotid artery, middle cerebral artery) were analyzed; they underwent MT with an aspiration catheter and/or stent retriever systems at the neurovascular center of a German university hospital from January 2014 to October 2018. At our center, MT is delivered 24/7 by 5 experienced neurointerventionalists. The study included all patients with intention to perform interventional thrombolysis in order to analyze any occurring circumstances leading to unsuccessful recanalization.

**Fig. 1:**
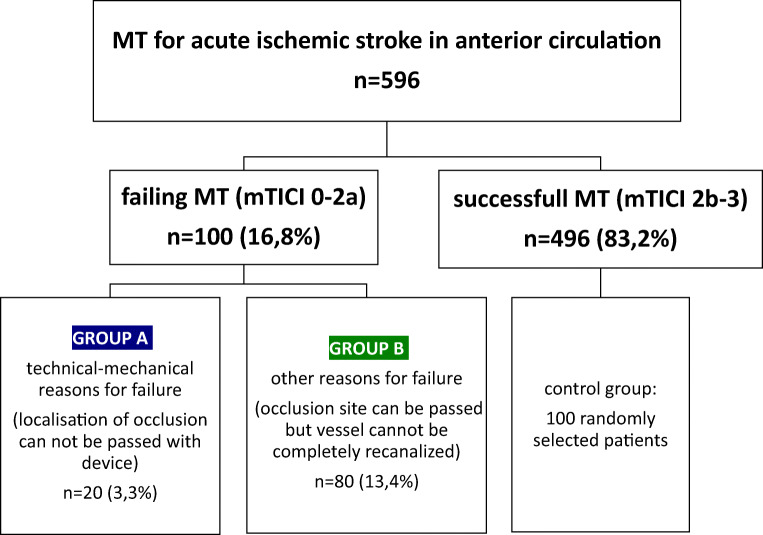
Selection process and subgroups’ definition

In our study, failure was defined as 0, 1, or 2a on the mTICI scale. The mTICI scale is a well investigated angiographic score for measuring recanalization success [11]. Figure 1 provides an overview of the selection process.

First, treatment protocols of unsuccessful MTs were investigated to find out the specific reasons of failure. Next, they were analyzed for interventional progress in order to identify the exact step when MT had failed.

Patients were analyzed for demographics, medical history including long-term medication, localization of the occlusion, severity of symptoms measured by the NIHSS [12], initial blood laboratory, and acute therapy. In addition, elapsed time from symptom onset to IVT and to interventional treatment were recorded, as well as intervention duration.

Additionally, patients with unsuccessful recanalization are divided into two subgroups, as indicated in Fig. 1.

- Group A: Patients with technical-mechanical reasons for failure (occlusion site cannot be passed with device).
- Group B: Patients with other reasons for failure (occlusion site can be passed but vessel cannot be completely recanalized).

The failing group in total and the two subgroups A and B were compared to a randomly selected group of 100 patients who underwent successful MT (mTICI 2b, 2c, and 3) in the same period at our center.

Data was recorded from treatment protocols, the angiographic system, and medical reports at discharge. Clinical data, especially examination results at discharge or later, was not collected at any time, since our study focused on angiographic parameters.

### Statistics

Absolute and relative frequencies are given for categorical data, median and IQR, or mean and SD for metric variables, as indicated. Comparisons of the group of successful interventions with the group of failing interventions and subgroups A and B were made using the Wilcoxon Mann-Whitney *U* test for quantitative variables and with the Fisher’s exact test and *X*_2_ test for qualitative variables, as appropriate. Significant lever was set at 5%, with a *p* value ≤ 0.05 considered as significant.

Statistical analysis was performed with SPSS, Version 25.0 (IBM Corporation, Armonk, New York, USA).

### Intervention procedure

MT was performed immediately after CT and CT angiography imaging under the following conditions: (1) acute ischemic stroke with NIHSS > 3 or severe isolated neurological deficit (aphasia, hemianopsia, isolated paresis of one limb), (2) large vessel occlusion with corresponding neurological deficits, (3) exclusion of hemorrhage, and (4) absence of any other individual contraindications for thrombectomy.

All the interventions were performed under general anesthesia and endotracheal intubation on a biplane angiography unit (Siemens Axiom Artis, Siemens Healthcare, Erlangen, Germany). The institution standard for occlusion of the anterior circulation consists of femoral approach with a 6F sheath (Cook Group Inc., Bloomington, Indiana, USA), which is placed as distal as possible in the internal carotid artery. Radial vessel access is very rare (1 of 100 in the failing group), and direct carotid puncture is currently not performed at our center. After microwire-assisted placement of the microcatheter, the microwire was exchanged for a thrombectomy device, and a stent retriever was deployed in the occluded vessel distal of the target lesion. Stent-retriever thrombectomy was executed with manual aspiration. If the intervention failed, another run was performed and/or material was changed. The mean number of maneuvers in total was 2.9 ± 2.0 (mean ± SD), maximum 9.

In some cases, especially proximal occlusions of large vessels, aspiration-first thrombectomy was carried out, while in other cases, a microcatheter was not used due to access difficulties. The choice of method and materials was made by the neurointerventionalist as well as the decision to terminate the procedure and to perform a rescue technique as permanent stenting or intraarterial tissue plasminogen activator. At the end of the intervention, post-interventional, the mTICI scale score was recorded.

## Results

Between January 2014 and October 2018, 100 of the 596 (16.8%) interventions failed according to our definition: 49 patients had a post-interventional mTICI scale score of 0, 18 had an mTICI scale score of 1, and 33 had an mTICI scale score of 2a. Table 1 gives an overview of failing interventions during the years 2014 until 2018.

**Table 1:** Failing interventions from 2014 until 2018 by years

	Number of interventions	mTICI 0	mTICI 1	mTICI 2a	% failing
2014	84	7	4	3	16.7%
2015	102	8	6	12	25.4%
2016	135	13	2	9	17.8%
2017	141	10	3	5	12.8%
01–10/2018	134	11	3	4	13.4%
2014–2018	596	49	18	33	16.8%

### Demographic data

As shown in Table 2, there was no significant difference in age or sex. The median age was 73.4 ± 12.8 years in the successful group and 71.1 ± 12.9 in the failing group. In subgroup B, patients seem to be slightly younger (70.1 ± 13.3 years), without the difference being significant. A small female predominance (*n* = 60; 60.0%) can be observed in the successful group, while all the other groups are almost equal in sex distribution (45.0–55.0%).

**Table 2:** Demographic parameters

	Success *n* = 100	Failure *n* = 100	*p*	Group A *n* = 20	*p*	Group B *n* = 80	*p*
Sex							
Female (%)	60 (60.0)	47 (47.0)	0.089^A^	9 (45.0)	0.227^A^	38 (47.5)	0.100^A^
Male (%)	40 (40.0)	53 (53.0)	0.089^A^	11 (55.0)	0.227^A^	42 (52.5)	0.100^A^
Age [years] (*X̅* ± SD)	73.4 ± 12.8	71.1 ± 12.9	0.132^B^	74.9 ± 10.8	0.972^B^	70.1 ± 13.3	0.078^B^

*A*, Fisher’s exact test; *B*, Mann-Whitney *U* test; *X̅*, mean; *SD*, standard deviation

### Interventional progress and material

**Fig. 2:**
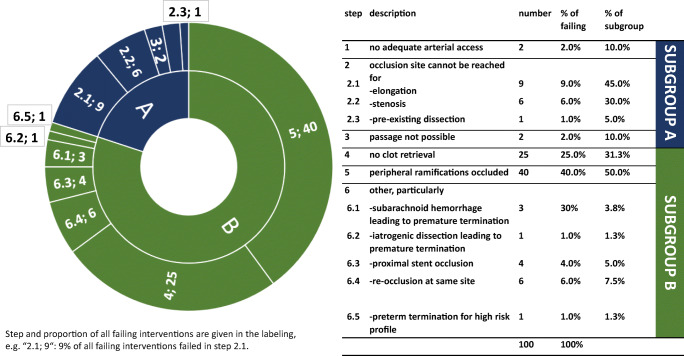
Sunburst diagram for interventional progress

Intervention was divided into 6 steps, as shown in Fig. 2.

In 20 of the 100 patients, it was not possible to reach and/or pass the occlusive lesion (steps 1–3 in Fig. 2, technical-mechanical reasons for failure). Most of them had elongation (9/20, 45.0%) or stenosis (6/20; 30.0%), which could not be passed with the endovascular device. In 2 cases (10.0%), femoral puncture was not possible, and in 1 case (5.0%), a carotid dissection was the reason for stroke symptoms. Thrombus passage with a microcatheter or microwire was not possible in 2 cases (10.0%). These 20 patients form group A, following the subgroups’ definition above.

In 80 of the 100 patients, the occluded site could be reached and/or passed, but the vessel could not be recanalized successfully (steps 4–6 in Fig. 2, true stent retriever failure). Those 80 patients form group B for the subgroup analysis. In most cases, peripheral ramifications remained occluded after the intervention (40/80, 50.0%) or no clot material could be removed at all (25/80, 31.3%). Ten patients suffered from a re-occlusion, 6 (7.5%) at the same site and 4 (5.0%) more proximal at a stent in the carotid artery that was placed due to a tandem lesion. Subarachnoid hemorrhage (3/80, 3.8%) and iatrogenic dissection (1/80, 1.3%) leading to premature termination are rare. In 1 case (1.3%), intervention was terminated for a high-risk profile (distal occlusion and difficult vascular anatomy with high risk of vessel perforation).

During the study period from January 2014 to October 2018, several types of stent retrievers and catheter systems were used in the group of failing interventions: 104 stent retrievers, including 60 Solitaires (Covidien, Mansfield, Massachusetts, USA), 15 ERICs (MicroVention, Tustin, California, USA), 16 embo-trap (Neuravi, Galway, Ireland), 12 pRESET (Phenox, Bochum, Germany), and 1 TREVO (Stryker, Kalamazoo, Michigan, USA). Furthermore, 92 intermediate catheters were used: 57 Sofia Intermediate Catheters (MicroVention, Tustin, CA, USA), 32 ACE or MAX Reperfusion Catheters (Penumbra, Alameda, CA, USA), 1 Neurobridge Intermediate Catheter (Acandis, Pforzheim, Germany), and 1 Envoy Guiding Catheter (DePuy Synthes Codman Neuro, Raynham, MA, USA).

Evaluation of the 100 failing interventions produces the following results: In 21 cases, no maneuver at all was possible, for example, due to access problems. In 7 , a stent retriever-alone maneuver and in 6 cases an aspiration-alone maneuver was performed, while in the 66 remaining cases, a combination of aspiration and stent retriever withdrawal was conducted. In 9 cases, an aspiration catheter was changed during the intervention; in 29 cases, a change of stent retriever was conducted, and in one case, a stent retriever was changed twice.

In 19 patients, balloon angioplasty was performed during the intervention; in 14, a stent was implanted.

### Comparisons of medical history include long-term medication, localization of the occlusion, severity of symptoms, initial blood laboratory, and acute therapy

All the characteristics of failing and successful intervention groups are given in Table 3.

**Table 3:** Patient-related parameters (*p* values are given for a comparison of the group of failing interventions with the successful group and for a comparison of subgroups A and B with the successful group)

	Success *n* = 100	Failure *n* = 100	*p*	Group A *n* = 20	*p*	Group B *n* = 80	*p*
Nicotine abuse (%)	22 (22.0)	24 (24.0)	0.876^A^	6 (30.0)	0.562^A^	18 (22.5)	1.000^A^
Coronary heart disease/myocardial infarction (%)	25 (25.0)	22 (22.0)	0.739^A^	7 (35.0)	0.409^A^	15 (18.8)	0.369^A^
Atrial fibrillation (%)	52 (52.0)	39 (39.0)	0.088^A^	10 (50.0)	1.000^A^	29 (36.3)	0.037^A^
Diabetes mellitus (%)	18 (18.0)	17 (17.0)	1.000^A^	4 (20.0)	0.761^A^	13 (16.2)	1.000^A^
Heart failure (%)	8 (8.0)	14 (14.0)	0.258^A^	2 (10.0)	0.672^A^	12 (15.0)	0.157^A^
Hyperlipoproteinemia (%)	27 (27.0)	27 (27.0)	1.000^A^	6 (30.0)	0.788^A^	21 (26.2)	1.000^A^
Hypertension (%)	74 (74.0)	72 (72.0)	0.874^A^	16 (80.0)	0.778^A^	56 (70.0)	0.616^A^
Chronic kidney disease (%)	8 (8.0)	8 (8.0)	1.000^A^	2 (10.0)	0.672^A^	7 (8.8)	1.000^A^
Patent foramen ovale (%)	2 (2.0)	5 (5.0)	0.445^A^	0 (0.0)	1.000^A^	5 (6.3)	0.244^A^
Endocarditis (%)	1 (1.0)	0 (0.0)	1.000^A^	0 (0.0)	1.000^A^	0 (0.0)	1.000^A^
Peripheral arterial occlusive disease (%)	4 (4.0)	10 (10.0)	0.164^A^	4 (20.0)	0.026^A^	6 (7.5)	0.343^A^
Autoimmune disease^#^ (%)	2 (2.0)	4 (4.0)	0.683^A^	0 (0.0)	1.000^A^	4 (5.0)	0.409^A^
Tumors^+^ (%)	6 (6.0)	7 (7.0)	1.000^A^	1 (5.0)	1.000^A^	6 (7.5)	0.768^A^
Coagulation disorder^~^ (%)	1 (1.0)	6 (6.0)	0.118^A^	0 (0.0)	1.000^A^	6 (7.5)	0.046^A^
Stroke/TIA in history (%)	19 (19.0)	16 (16.0)	0.710^A^	5 (25.0)	0.547^A^	11 (13.8)	0.423^A^
Thrombosis/lung embolism in history (%)	2 (2.0)	4 (4.0)	0.683^A^	1 (5.0)	0.424^A^	3 (3.8)	0.479^A^
Low molecular weight heparin (%)	6 (6.0)	3 (3.0)	0.498^A^	2 (10.0)	0.619^A^	1 (1.3)	0.134^A^
Unfractionated heparin (%)	3 (3.0)	1 (1.0)	0.621^A^	0 (0.0)	1.000^A^	1 (1.3)	0.630^A^
Coumarins (%)	10 (10.0)	9 (9.0)	1.000^A^	1 (5.0)	0.689^A^	8 (10.0)	1.000^A^
New oral anticoagulants (%) Dabigatran (%) Edoxaban (%) Apixaban (%) Rivaroxaban (%)	8 (8.0) 2 (2.0) 1 (1.0) 2 (2.0) 3 (3.0)	6 (6.0) 0 (0.0) 0 (0.0) 5 (0.0) 1 (1.0)	0.783^A^ 0.497^A^ 1.000^A^ 0.445^A^ 0.621^A^	1 (5.0) 0 (0.0) 0 (0.0) 1 (5.0) 0 (0.0)	1.000^A^ 1.000^A^ 1.000^A^ 0.424^A^ 1.000^A^	5 (6.3) 0 (0.0) 0 (0.0) 4 (5.0) 1 (1.3)	0.776^A^ 0.503^A^ 1.000^A^ 0.409^A^ 0.630^A^
Ace-inhibitors (%)	30 (30.0)	27 (27.0)	0.754^A^	8 (40.0)	0.435^A^	19 (23.8)	0.401^A^
AT1-receptor antagonists 8%)	23 (23.0)	19 (19.0)	0.603^A^	4 (20.0)	1.000^A^	15 (18.8)	0.582^A^
Betablockers (%)	61 (61.0)	55 (55.0)	0.474^A^	12 (60.0)	1.000^A^	43 (53.8)	0.364^A^
Diuretics (%)	35 (35.0)	43 (43.0)	0.310^A^	9 (45.0)	0.450^A^	34 (42.5)	0.355^A^
Calcium channel blockers (%)	27 (27.0)	19 (19.0)	0.239^A^	3 (15.0)	0.397^A^	16 (20.0)	0.296^A^
Nitrates (%)	8 (8.0)	1 (1.0)	0.035^A^	1 (5.0)	1.000^A^	0 (0.0)	0.009^A^
Statins (%)	31 (31.0)	30 (30.0)	1.000^A^	10 (50.0)	0.124^A^	20 (25.0)	0.409^A^
Metamizole (%)	10 (10.0)	9 (9.0)	1.000^A^	2 (10.0)	1.000^A^	7 (8.8)	0.804^A^
Ibuprofen (%)	2 (2.0)	4 (4.0)	0.682^A^	0 (0.0)	1.000^A^	4 (5.0)	0.409^A^
Acetylsalicylic acid (%)	21 (21.0)	31 (31.0)	0.146^A^	9 (45.0)	0.044^A^	22 (27.5)	0.380^A^
Clopidogrel (%)	4 (4.0)	8 (8.0)	0.373^A^	3 (15.0)	0.090^A^	5 (6.3)	0.514^A^
Thyroxine (%)	17 (17.0)	16 (16.0)	1.000^A^	4 (20.0)	0.751^A^	12 (15.0)	0.839^A^
Glucocorticoids (%)	2 (2.0)	5 (5.0)	0.445^A^	2 (10.0)	0.129^A^	3 (3.8)	0.657^A^
Lesion in internal carotid artery (%)	40 (40.0)	42 (42.0)	0.886^A^	13 (65.0)	0.050^A^	29 (36.3)	0.646^A^
Lesion in middle cerebral artery. M1 (%)	49 (49.0)	39 (39.0)	0.200^A^	3 (15.0)	0.006^A^	36 (45.0)	0.653^A^
Lesion in middle cerebral artery M2 (%)	11 (11.0)	19 (19.0)	0.165^A^	4 (20.0)	0.274^A^	15 (18.8)	0.200^A^
Right hemisphere (%)	43 (43.0)	39 (39.0)	0.666^A^	11 (55.0)	0.338^A^	28 (35.0)	0.287^A^
Left hemisphere (%)	57 (57.0)	61 (61.0)	0.666^A^	9 (45.0)	0.338^A^	52 (65.0)	0.287^A^
Initial NIH-Stroke-Scale (M and IQR)	15 (11–19)	14 (10–19)	0.578^B^	14 (10–17)	0.457^A^	15 (10–20)	0.723^B^
Intravenous thrombolysis (%)	51 (51.0)	35 (35.0)	0.032^A^	7 (35.0)	0.226^A^	28 (35.0)	0.035^A^
Thrombolysis-intervention time [min] (M and IQR)	90 (41–151)	118 (47–161)	0.351^B^	139 (79–228)	0.142^A^	102 (46–152)	0.652^B^
Onset-needle time [min] (M and IQR)	90 (73–126)	91 (77–117)	0.413^B^	95 (80–150)	0.793^B^	91 (75–132)	0.404^B^
Onset-groin-puncture time [min] (M and IQR)	158 (125–198)	189 (134–261)	0.608^B^	271 (151–333)	0.155^B^	200 (140–270)	0.973^B^
Onset-end-of-intervention Time [min] (M and IQR)	194 (145–239)	236 (220–301)	0.055^B^	308 (188–375)	0.199^B^	272 (202–318)	0.085^B^
Intervention duration [min] (M and IQR)	23 (14–36)	50 (36–90)	0.000^B^	27.5 (23–52)	0.665^B^	58 (44–73)	0.000^B^
Stroke of unknown onset (%)	20 (20)	26 (26)	0.401^A^	6 (30.0)	0.374^A^	20 (25.0)	0.473^A^
C-reactive protein [mg/l] (M and IQR)	4.5 (1.9–14.6)	5.3 (2.4–15.4)	0.342^B^	6.1 (1.7–24.1)	0.420^B^	5.1 (2.5–12.1)	0.431^B^
White blood cell count [×10^9^/l] (*X̅* ± SD)	10.6 ± 4.2	10.4 ± 5.3	0.284^B^	10.1 ± 4.3	0.576^B^	9.2 ± 3.9	0.303^B^
INR (M and IQR)	1.06 (0.99–1.14)	1.04 (0.97–1.10)	0.276^B^	1.06 (1.01–1.11)	0.719^B^	1.03 (0.97–1.10)	0.153^B^
aPTT [s] (M and IQR)	24 (23–27)	24 (22–27)	0.639^B^	25 (22–28)	0.899^B^	24 (23–27)	0.546^B^
Platelet count [×10^9^/l] (M and IQR)	228 (186–274)	223 (160–277)	0.258^B^	199 (142–276)	0.108^B^	227 (170–277)	0.500^B^

*A*, Fisher’s exact test; *B*, Mann-Whitney *U* test; *M*, median, *IQR*, interquartile range; *X̅*, mean; *SD*, standard deviation

^#^Vasculitis, Hashimoto thyroiditis, Crohn’s disease, rheumatoid arthritis, myasthenia gravis. No significant difference in any of the subcategories

^+^Active tumor disease: small cell lung cancer, non-small cell lung cancer, colorectal carcinoma, mammary carcinoma, non-Hodgkin’s lymphoma, chronic lymphocytic leukemia, plasmocytoma, malignant melanoma, thyroid cancer, transitional cell carcinoma of the urinary tract, oropharyngeal squamous cell carcinoma, meningeoma, prostate cancer, cancer of unknown primary. No significant difference in any of the subcategories

^~^Heterozygous and homozygous G20210A-mutation, antiphospholipid syndrome, factor V Leiden mutation, protein c/s-deficiency, thrombotic thrombocytopenic purpura, paraneoplastic coagulation disorder. No significant difference in any of the subcategories

In our study, IVT before intervention is associated with recanalization success. In the successful group (SG), 51.5% of all patients were treated with intravenous tissue plasminogen activator, while in the failure group (FG), only 35.0% received this medication (*p* = 0.032). Furthermore, there was a statistical correlation between the long-term intake of nitrates and a successful intervention (SG = 8.0% vs. FG = 1.0%; *p* = 0.035).

Intervention duration was significant longer in failing interventions (SG = 23 (14–36) min vs. FG = 50 (36–90) min; *p* = 0.000).

Regarding coagulation therapy with coumarins or direct oral anticoagulants, no significant difference was found in any of the groups. Time factors related to symptom onset, to IVT, and to intervention do not seem to play a role in the success of recanalization. Laboratory parameters for inflammatory response and coagulation were comparable in all groups (see Table 2).

### Subgroup analysis

In addition to the results given for the total failing group, there are interesting findings for the two subgroups:

Compared to the successful group, in group A (A) there is a strong association between failing intervention and PAOD (SG = 4.0% vs. *A* = 20.0%; *p* = 0.026) as well as the intake of acetylsalicylic acid (SG = 21.0% vs. *A* = 45.0%; *p* = 0.044). In group A, lesions are significantly more often located in the internal carotid artery (SG = 40.0% vs. *A* = 65.0%; *p* = 0.050) and more rarely in the middle cerebral artery, segment M1 (SG = 49.0% vs. *A* = 15.0%; *p* = 0.006).

In group B (*B*), atrial fibrillation (AF) is associated with recanalization success (SG = 52.0% vs. *B* = 36.3%; *p* = 0.037), whereas coagulation disorders are associated with poor results (SG = 1.0% vs. *B* = 7.5%; *p* = 0.046). Coagulation disorders particularly were defined as heterozygous (1 patient in B) and homozygous G20210A mutation (0 patients), antiphospholipid syndrome (2 patients, 1 in SG and 1 in B), factor V Leiden mutation (2 patients in B), protein c/s deficiency (1 patient in B), thrombotic thrombocytopenic purpura (0 patients), or paraneoplastic coagulation disorder (1 patient in B).

## Discussion

Several retrospective investigations concerning failing thrombectomy were published in recent months [13, 14]. In our study, we were able to detect success rates of 83.2% at our center. We analyzed interventions from 2014 to 2018. In this period, major improvements of material and methods were made [15]. We found a peak of failing interventions in the year 2015 with many recanalization results of mTICI 2a. When having a second look at the angiographic reports, no reason therefore could be found, especially no discrepancies in material or interventionalist. In 2014 and from 2016 to 2018, the success rate at our center is in the range of 83–86%. Recent literature comes to similar success rates of around 80–89% [6, 16], while older reviews indicate success rates of 80% [17].

A major problem when comparing different studies is the heterogeneous definition of success. In particular, a reperfusion result of mTICI 2a is sometimes regarded as success, sometimes as failure [6, 13, 14, 18, 19].

In 20% of the failing interventions, the thrombus could not be reached or passed with the device while in 80% recanalization failed due to other reasons. Literature reports similar data [13]. For patients failing due to mechanical reasons, direct carotid puncture or surgical vascular access, which is currently not performed at our center, could be a therapeutic option in certain cases. Direct carotid puncture is a high-risk procedure, but recent literature reports successful recanalization in 8/11 reported cases, where a femoral approach had failed [20]. Further options for access problems, e.g., neck extension to stretch tortuous or elongated vessels should be investigated in upcoming studies [21].

In addition to procedural aspects, we analyzed patients’ medical history. IVT is associated with improved recanalization rates. This can be explained by the pharmacologic mechanism: the clot is dissolved from the outer edge and can therefore be accessed by the mechanical device easier [22]. Another explanation could be, that small fragments of the initial thrombus are dissolved during the intervention and do not occlude distal branches. Several other studies come to similar findings when comparing patients with and without IVT for recanalization success, as shown in a recent review by Pan et al. [23]. It was to be expected that vascular diseases such as PAOD influence MT in a negative way. Coagulation disorders also are a challenge for interventional treatment, especially because of a high rate of re-occlusion during the intervention. We were able to prove a significant correlation between PAOD and failing intervention, as well as between coagulation disorders and unsuccessful thrombectomy.

In the group of patients where thrombus could be reached but the vessel remained occluded, AF is significantly less frequent. Recent literature also shows inconclusive findings concerning this aspect [24]. There are studies and histological analysis that come to a similar result [25, 26], while other investigations indicate the opposite: cardiogenic thrombi seem to have a higher proportion of fibrin compared to other stroke etiologies, associated with worse interventional recanalization rates [27–29]. More studies on thrombus etiology and thrombus composition must be performed.

It is not clear why there is a strong association of intake of NO donors and reperfusion success. Recent studies investigated NO donors as symptomatic treatment for acute ischemic stroke, but not as a medication to improve recanalization success [30]. Further studies are required to find out whether the result is an artifact or not.

There was no difference in any kind of anticoagulation therapy with coumarins, NOAC, or heparins regarding recanalization success. This is of special interest since many future patients will take NOACs instead of coumarins and we do not know the effects of NOACs on thrombus characteristics.

In the subgroup of failing interventions, where target lesion could be reached with the device, techniques should be improved, and new material should be developed. For example, recent studies suggest a proximal balloon occlusion during stent retriever withdrawal to protect the distal vessel from thrombus fragments that have sheared off [31]. As a rescue therapy, permanent stenting or intraarterial thrombolysis as well as administration of tirofiban are options worth considering [32–34]. In certain cases, creativity on the part of the interventionalist can lead to success: there are case reports of dual-use of stent retrievers for refractory clots involving vessels’ bifurcation [35]. In general, more evidence for the question of how to treat patients with failing thrombectomy is required.

Since all patients in both groups were under general anesthesia during the intervention and we investigated the recanalization itself, we did not analyze anesthesiologic medication or blood pressure during the intervention. In literature, there is evidence for anesthesiologic management influencing outcome, but not recanalization results [36–39].

### Limitation

It should be noted that only angiographic data and no clinical parameters concerning patient outcome were collected. Nevertheless, there is a strong correlation between successful reperfusion and patients’ outcome, as shown in recent literature [9, 10, 19, 40]. The study cohort is the same size as in other comparable studies. Another limitation is the retrospective study design.

## Conclusion

There are two main reasons for failing MT:

Major problems of failing thrombectomy, on the one hand, include a difficult vascular situation affecting interventional treatment in 20% of all failing interventions. The target lesion cannot be reached and/or passed with the device. Especially in those patients, PAOD is a common previous disease.

In the other 80% of all failing interventions, thrombus can be passed with the device, but the occluded vessel cannot be recanalized. Coagulation disorders are associated with poor results, whereas atrial fibrillation, intake of NO donators, and administration of intravenous thrombolysis are associated with higher recanalization rates.

Future research on improving recanalization rates should concentrate on the two key areas of access problems and true stent retriever failure.
